# An Experimental Study of the Acoustic Signal Characteristics of Locked-Segment Damage Evolution in a Landslide Model

**DOI:** 10.3390/s24154947

**Published:** 2024-07-30

**Authors:** Xing Zhu, Hui Chen, Zhanglei Wu, Shumei Yang, Xiaopeng Li, Tiantao Li

**Affiliations:** 1 State Key Laboratory of Geohazard Prevention and Geoenvironment Protection, Chengdu University of Technology, Chengdu 610059, China; zhuxing15@cdut.edu.cn (X.Z.);; 2 College of Computers and Cyber Security, Chengdu University of Technology, Chengdu 610059, China; 3 Sichuan Engineering Technology Research Center of Industrial Internet Intelligent Monitoring and Application, Chengdu University of Technology, Chengdu 610059, China; 4 The Geomathematics Key Laboratory of Sichuan Province, Chengdu University of Technology, Chengdu 610059, China; 5 Powerchina Chengdu Engineering Corporation Limited, Chengdu 610050, China

**Keywords:** three-section landslide, locking section, video image, micro-seismic signal, acoustic emission

## Abstract

Three-section landslides are renowned for their immense size, concealed development process, and devastating impact. This study conducted physical model tests to simulate one special geological structure called a three-section-within landslide. The failure process and precursory characteristics of the tested samples were meticulously analyzed using video imagery, micro-seismic (MS) signals, and acoustic emission (AE) signals, with a focus on event activity, intensity, and frequency. A novel classification method based on AE waveform characteristics was proposed, categorizing AE signals into burst signals and continuous signals. The findings reveal distinct differences in the evolution of these signals. Burst signals appeared exclusively during the crack propagation and failure stages. During these stages, the cumulative AE hits of burst signals increased gradually, with amplitude rising and then declining. High-amplitude burst signals were predominantly distributed in the middle- and high-frequency bands. In contrast, cumulative AE hits of continuous signals escalated rapidly, with amplitude monotonously increasing, and high-amplitude continuous signals were primarily distributed in the low-frequency band. The emergence of burst signals and high-frequency AE signals indicated the generation of microcracks, serving as early-warning indicators. Notably, the early-warning points of AE signals were detected earlier than those of video imagery and MS signals. Furthermore, the early-warning point of burst signals occurred earlier than those of continuous signals, and the early-warning point of the classification method preceded that of overall AE signals.

## 1. Introduction

The majority of rock landslides are highly sudden, destructive, and widespread geological disasters. The overall stability of most large rock landslides is controlled by the key rock masses in the locking section on weak structural planes or shear zones. Such landslides are referred to as locking-section landslides [1]. Among them, the three-section landslide is a typical type of locking-section landslide, which is common in the western regions of China [2]. The generic mechanism of the three-section landslide involves sliding, tension cracking, and shearing. In essence, creep occurs on the low inclined cataclinal structural planes, followed by the appearance of tensile cracks at the slope’s rear edge, ultimately resulting in damage to the central locking section [2]. Globally, the economic losses and casualties caused by rock landslides, particularly three-section landslides, are increasing [3]. The locking section is a crucial load-bearing component of the slope, and its strength and deformation determine the overall stability of the slope [4,5,6,7]. Therefore, a deeper understanding of the failure mechanisms and early-warning indicators of the locking section is imperative.

In recent years, researchers have conducted numerous studies on rock landslides involving the locking section, focusing on failure mechanisms and mechanical properties of rock. Huang et al. [8] and Pan et al. [9] classified rock landslides with a locking section by studying essential characteristics of multiple large-scale catastrophic landslides and elucidated the sliding mechanisms of the locking section. From the perspective of rock’s mechanical properties, many scholars have discussed the influence of different angles of joints [10,11] and varying bridge lengths [12] on the failure mechanism of the locking section. Although these studies have shed light on the failure mechanism of three-section landslides, significant challenges in early warning persist due to the inconspicuous macroscopic failure deformation.

The deformation or failure of rock involves the generation and propagation of cracks under external loads, releasing elastic waves known as AE activity in the laboratory tests [13,14,15,16,17]. Acoustic emission (AE) signals are widely utilized for their capability to monitor failure behavior [18]. Currently, AE technology has become an innovative and powerful tool for monitoring rocks, slopes and tunnels, especially in the study of the correlation between slope instability and AE behavior [19,20,21]. Kumar et al. [22] conducted physical experiments on artificial soil slopes and demonstrated that the threshold deformation rate can be achieved in the engineering design of a landslide early-warning system based on AE technology. Deng et al. [23] developed a complete AE monitoring system and verified its practical performance at 22 landslide sites. These studies provide a reliable basis for the landslide early-warning system. Further investigation into AE phenomena during the fracture process of the locking section is warranted. Moradian et al. [24] established the relationship between cracking levels in brittle rocks and cumulative AE hits and the energy of AE signals. Additionally, Moradian et al. [24] analyze the relationship between shear cracking behavior and AE by examining count and energy parameters, highlighting the AE monitoring’s effectiveness in interpreting the shear cracking process of in situ discontinuities. While these studies provide robust support for understanding rock damage mechanisms, AE characteristic parameters offer only a simplistic statistical description of the waveform, lacking in-depth information [25].

Numerous scholars have delved into the AE waveform to reveal deeper insights into the rock’s damage process. Schiavi et al. [26] and Patricia Rodríguez et al. [27] interpreted the behavior of the main frequency components of AE signals. Wang et al. [28] analyzed the frequency–amplitude distributions of AE signals throughout the failure process. They observed a noticeable increase in the amplitude of AE signals as stress increments, further accompanied by low-frequency components. More recently, researchers have delved into the sub-frequency characteristics. Mei et al. [29] classified AE signals based on both main frequencies and sub-frequencies characteristics, exploring their variations. However, focusing solely on the overall features of AE may overlook crucial information regarding rock fracture. Therefore, some scholars have categorized AE signals into burst signals and continuous signals based on their waveform characteristics, investigating their behavior [30]. Nevertheless, comprehensive analyses of these two types of AE signals during the process of rock fracture remain limited.

The AE characteristics of rock deformation and failure have been extensively studied in laboratory tests, while micro-seismic (MS) signals monitoring is commonly employed to predict the rock failure modes in engineering applications. Leśniak [31] and Pastén et al. [32] have analyzed rock failure mechanisms and stability by examining the spatial and temporal evolution of MS events. Calder and Semadeni [33], as well as Xiao et al. [34], have studied the frequency characteristics of MS events and proposed precursor indicators for rock failure. Furthermore, rock failure typically initiates with the formation of microcracks, followed by macroscopic crack propagation [35]. With advancements in digital imaging technology, techniques such as high-speed photography [36], scanning electron microscope [37], infrared thermography [38], and computerized tomography [39,40] have been introduced for monitoring and early warning of rock mass failure. Consequently, digital image processing has become an essential technical method for studying crack propagation modes and failure patterns.

This research, based on the video image and characteristics data obtained from laboratory tests designed to replicate the geological structure of the three-section landslide, aims to explore the evolutionary patterns and precursory characteristics of MS and AE signals. The investigation focuses on event activity, event intensity, and frequency. Furthermore, a novel classification method is proposed to categorize AE signals into burst signals and continuous signals. The evolution characteristics of burst signals and continuous AE signals are further examined through analysis of AE characteristic parameters (AE hits) and waveform attributes (amplitude and frequency). The method is more suitable as a precursor warning for sudden landslide disasters than deformation monitoring indicators.

## 2. Experimental Methodology

### 2.1. Sample Preparation

**Figure 1:**
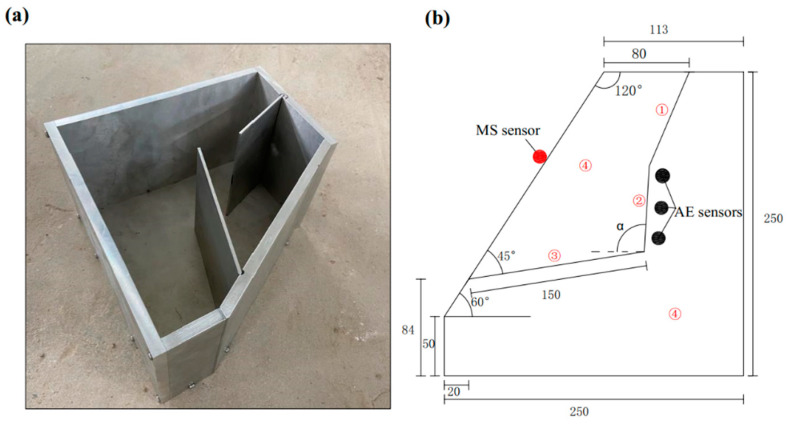
(**a**) Preprepared mold of tested samples, and (**b**) conceptual model of the locking section (unit: mm). 1—Tensile crack of the back; 2—Locking section in the middle; 3—Creep segment of the front; 4—Rock slope; α—The angles of the rock bridge.

**Figure 2:**
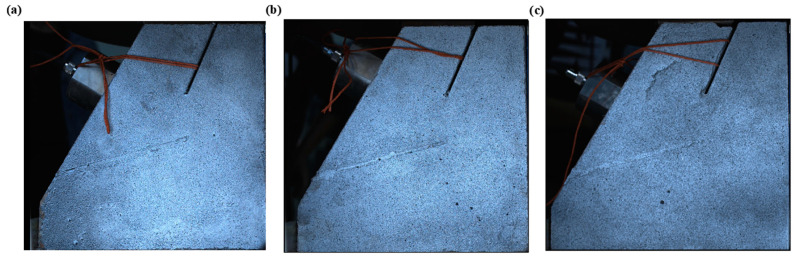
Photos of test samples: (**a**) sample #1; (**b**) sample #2; and (**c**) sample #3.

The geo-mechanical model tests of three-section landslides with different angles of rock bridge were designed and prepared. The tested samples were fabricated by casting concrete using pre-prepared molds. The angle of the rock bridge of the locking section, denoted as α in Figure 1b, is defined as the angle between the line extending from the end of the tensile crack to the end of the creep section and the horizontal direction. In Figure 2, the angles of the rock bridge are 70°, 90°, and 110°, respectively. The sample material consisted of cement, lime, sand, and water, with a mass ratio of 5:1:13:4. The physical model of the specimen, as depicted in Figure 1a, was divided into four parts. The size and shape of the slope model are illustrated in Figure 1b. After the mold was filled tightly, the concrete was cured and dried under natural conditions to form the sample.

### 2.2. Test Apparatus

In this study, a WA08 strain acceleration sensor was utilized to collect MS signal, with an acquisition frequency set as 1 kHz. Concurrently, AE signals were automatically detected and collected using a Micro-II type AE system by Physical Acoustics from Princeton in America. The main amplifier of the AE test system was set to a gain of 40 dB, with a threshold set at 40 dB. The sampling frequency was configured as 1 MHz, and each waveform was sampled with 1024 points per second. To ensure the accuracy of the test results, a layer of petroleum jelly was applied between each rock specimens and the AE sensors to maintain good connections. The test employed a YH1000 microcomputer-controlled pressure testing machine from Chengdu in China. The loading method employed was displacement control mode, with loading rate of 0.2 mm/min, and preloading was set at 1 kN. Finally, the failure termination criterion for the sample was set as a displacement of 5 mm.

### 2.3. Test Schemes

A high-speed camera was employed to record the fracture process of the tested samples. Additionally, an MS sensor and three AE sensors were installed on each sample, as illustrated in Figure 1b. Once the test commenced, the lower pressing plate of the specimen machine was gradually raised to allow the cushion block to contact with the upper pressing plate, initiating stress on the locking-section model. Upon reaching a preload of 1 kN, the corresponding MS and AE signals were synchronously recorded. Simultaneously, the loading system recorded the stress data until the stress decreased rapidly upon instability and the specimen destruction. Statistical analysis of the time of AE signals received from three sensors in each test was conducted in this study. Effective time points were selected from the numerous AE signals for reduced computational burden. Each sensor exhibited good consistency in AE data across each test. Therefore, the sensor data with the largest amplitude were selected for analysis at the effective time point. Due to data similarity across the three tests and space limitations, detailed analysis in this paper focused primarily on sample #1. The results of sample #2 and sample #3 were briefly discussed.

## 3. Results and Analysis

**Figure 3:**
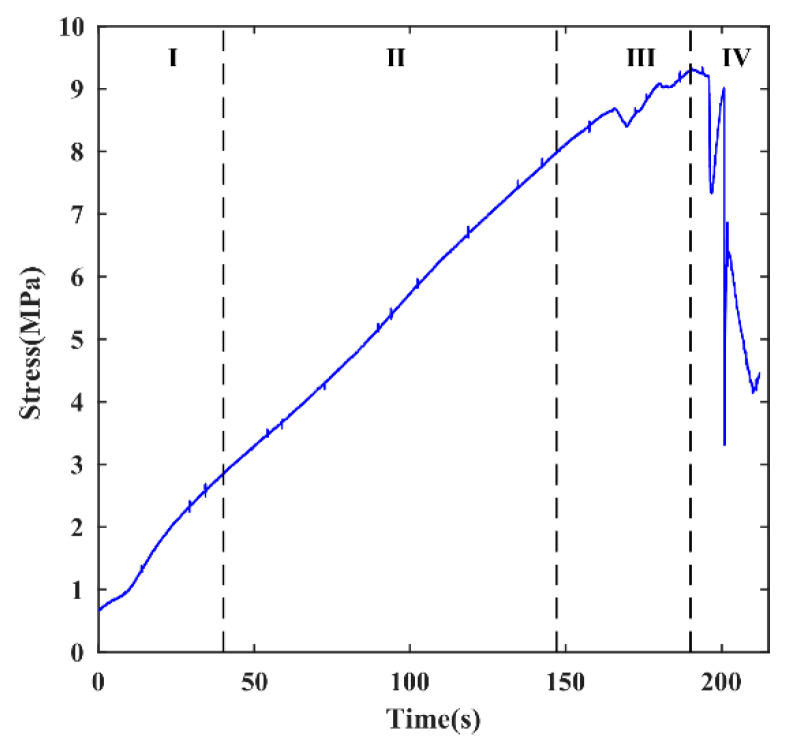
The relational curve between stress and time of sample #1.

The relationship between stress and time for sample #1 is depicted in Figure 3. The time–stress characteristics are similar to the axial deformation characteristics of rocks under uniaxial compression conditions. Therefore, with reference to the crack propagation stages commonly used in rock mechanics tests, the loading process of sample #1 was segmented into the following stages [41]:

Stage I: Compaction stage (0 to 40 s). This stage involves the mutual friction and compression of particles in the model under the applied load, resulting in an accelerated increase in the stress curve over time.

Stage II: Elastic deformation stage (40 to 147 s). During this stage, the time–stress curve that approximates a straight line, indicating a well-recoverable shear resistance within the locked section, is shown.

Stage III: Crack propagation stage (147 to 190 s). This stage shows an overall upward trend in the stress curve with multiple sudden drops, indicating the occurrence of large-scale microcracking events within the locked section due to shear compression from the upper load.

Stage IV: Rock instability and failure stage (190 to 212 s). Characterized by a significant drop in the stress curve, this stage signifies the complete loss of locking ability as the fractures in the locked section become fully connected, resulting in the complete release of energy.

For clarity, stages III and IV are collectively referred to as the crack propagation and failure stage.

### 3.1. Analysis of Video Image

**Figure 4:**
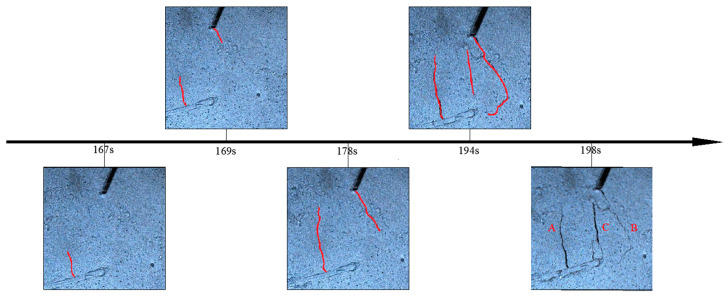
Video images of macrocracks on sample #1. The red lines in the figures indicated the nascent macroscopic cracks.

Video imaging serves as an effective method to observe the specimen’s failure process. Figure 4 illustrates the crack growth of sample #1. During stages I and II, the video imagery did not reveal any significant information [23]. Subsequently, three macrocracks, denoted as A, B, and C, respectively, emerged in the sample. The lower end of crack A appeared in the front part of the locking section at 167 s (0:02:47), with its propagation direction perpendicular to the creep segment. The upper end of crack B was generated at 169 s (0:02:49). With increasing load, crack A progressed upward while crack B extended downward, indicating alternating shear failure in the front and back of the locking segment. The primary crack C emerged at 194 s (0:03:14), propagating along the direction of the locked section. At 198 s, the main crack C caused relative slippage, resulting in instability and failure of the locking segment. The failure of the slope is delineated by cracks connection in the locking section. Considering the occurrence of macrocracks as the early-warning point of rock instability, this key point precedes the time of unstable failure by 27 s.

### 3.2. Analysis of MS Signal

#### 3.2.1. Activity and Intensity

**Figure 5:**
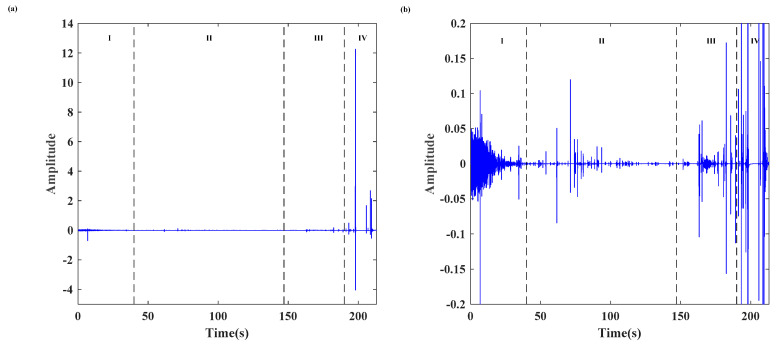
(**a**) The MS waveform, and (**b**) a partial enlargement of (**a**).

In this test, the sampling frequency of the MS signal is set at 1 kHz. Firstly, the MS signal is prefiltered using the wavelet-based de-noising method to mitigate noise interference in the experimental environment [42]. The density and amplitude of the MS signal waveform directly reflect the activity and intensity of the event, respectively. The time-domain waveform of the MS signal is presented in Figure 5. The typical evolutionary process of the MS signal and the corresponding failure process can be described in detail as follows.

(1) In the stage of compaction due to initial damage (stage I), the density and amplitude of the MS signal are small as the external pressure gradually compresses the pre-existing cracks, voids, and/or other defects.
(2) During the elastic deformation stage (stage II), the density and amplitude of the MS signal are lower than those in the previous stage (stage I), indicating insufficient force to induce the formation of new microcracks in the locking section under the current load level. Notably, a significant locking effect and stress accumulation along the locking section are observed due to the creeping segment at the front and the tensile crack at the back of the slope.

In the stage of cracks propagation and failure (stages III and IV), the density and amplitude of the MS signal increase, signifying intense activity within the specimen and gradual failure of the locked segment. Particularly after the main crack is generated, the high-amplitude MS signal emerges continuously. The maximum amplitude was 12.25 V at 198 s when the main crack caused relative slippage, indicating sudden brittle shear failure in the locking section and an instantaneous release of tremendous energy.

#### 3.2.2. Frequency

**Figure 6:**
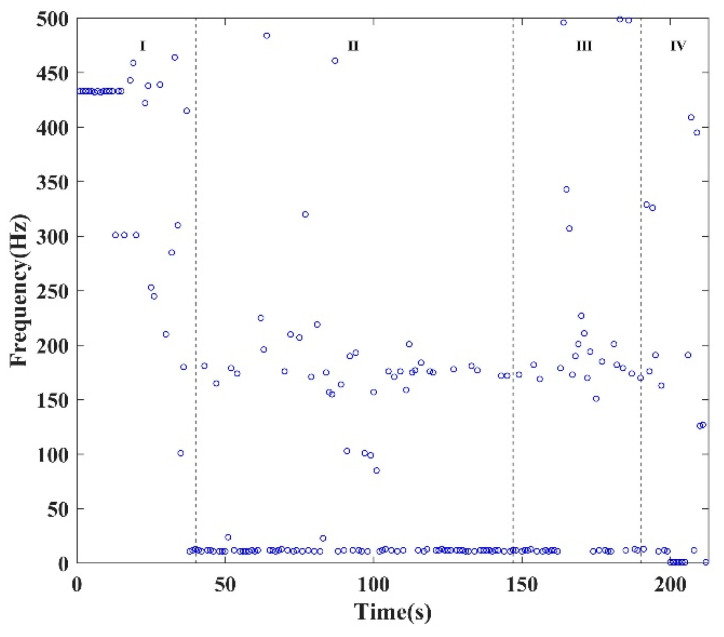
The variation in main frequencies of the MS.

The continuous MS signal, filtered and sampled at 1024 points per second, underwent Fast Fourier Transform (FFT) analysis to extract the main frequencies from the sampled MS events. The corresponding main frequencies were identified based on their definition, and Figure 6 illustrates the evolutionary process of the main frequencies during the failure process of the tested specimen. This process can be described in detail as follows.

(1) In the compaction stage due to initial damage (stage I), the main frequencies ranged from 250 to 500 Hz, gradually transitioning towards the low-frequency band.
(2) During the elastic deformation stage (stage II), the main frequencies of the intercepted MS signals were predominantly distributed below 250 Hz.
(3) In the crack propagation and failure stage (stages III and IV), high-frequency MS signals reappeared. At 163 s, the main frequency was 487.3 Hz. The resurgence of the high-frequency component signifies shear failure in the locking section and the emergence of microcracks appeared in the locking section. With increasing load, numerous microcracks in the locking section will expand and form macrocracks on the free face of the locking section. Thus, the reappearance of the high-frequency component serves as an early-warning point for rock instability. Notably, the first macrocrack appeared on the free face of the locking section under loading conditions at 167 s, while the time of the early-warning point of the MS signal was 163 s. Therefore, the time of the early-warning point of the MS signal precedes that of the video image.

### 3.3. Classification Method of AE Signals

**Figure 7:**
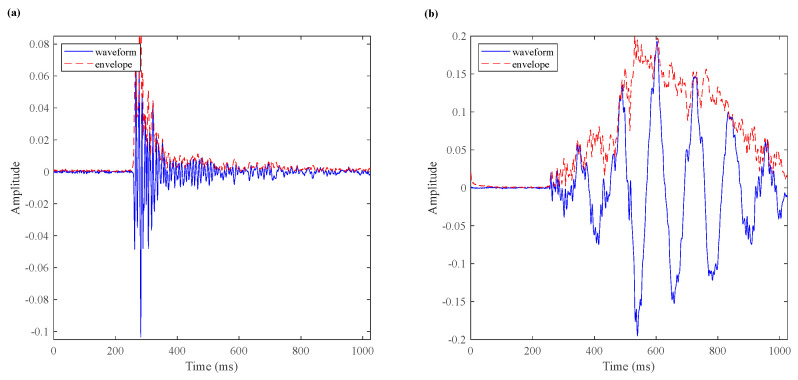
The waveform and its envelope: (**a**) typical burst signal, and (**b**) typical continuous signal.

AE signals are the elastic waves released in the deformation or failure process. Although both AE and MS sensors utilize the piezoelectric principle to convert vibration signals into electrical signals, the MS signals are mainly generated by stress release resulting from rock fractures or sliding. These signals are typically associated with macroscopic geological activities, rock deformations, and rock layer ruptures. On the other hand, AE signals are generated within small, localized areas of the material and are usually caused by internal stress, crack propagation, particle displacement, and other factors. Based on its time-domain morphology, AE signals can be classified into burst signals and continuous signals [43]. Burst signals are AE signals whose waveform is pulse waveform and can be separated in the time domain. In contrast, continuous signals do not have clear separations in time and typically indicate the simultaneous occurrence of numerous AE events [44]. The blue line in Figure 7a,b shows the typical burst signal waveform and continuous signal waveform, respectively. Tens of thousands of AE signals are released during the rock failure process. Efficiently classifying and extracting key information from these massive waveform data are crucial. The traditional classification methods rely on manual screening, which is time-consuming and prone to errors. Therefore, a scientific and efficient calculation method is urgently needed for automatic classification.

In this paper, a new classification method is proposed based on the characteristics of the AE waveform. The flow of the algorithm is as follows: (1) To fit the waveform and the red line in Figure 7a,b is the fitting envelope; (2) To find the peak of the envelope, that is, to find the peak (amplitude) and peak position (time); (3) Count the number of peaks whose peak value is greater than 50% of the maximum peak value, and the number is denoted by *N*; (4) If 1<N≤3, and these peaks are all adjacent, go to the next step. If N=1, it is a burst signal (algorithm end). Otherwise, it is a continuous signal (algorithm end); (5) Calculate the distance between its adjacent peaks, denoted by $L_{i}$, respectively. If $\forall L_{i}\leq 100,i=1,\ldots ,N-1$ (the distance is less than 0.1 ms), it is a burst signal. Otherwise, it is a continuous signal. 

Using this algorithm, AE signals were classified into burst signals and continuous signals. The distribution of AE signals across stages during the failure process is presented in Table 1. The table shows the total proportion of continuous signals is as high as 80.75%, indicating a scarcity of burst signals during the test. Importantly, burst signals were exclusively observed during the stages of crack propagation and failure (stages III and IV). Therefore, subsequent analyses will focus solely on burst signals starting from stage III.

## 4. Analysis of Classified AE Signals

### 4.1. AE Hits (Activity)

**Figure 8:**
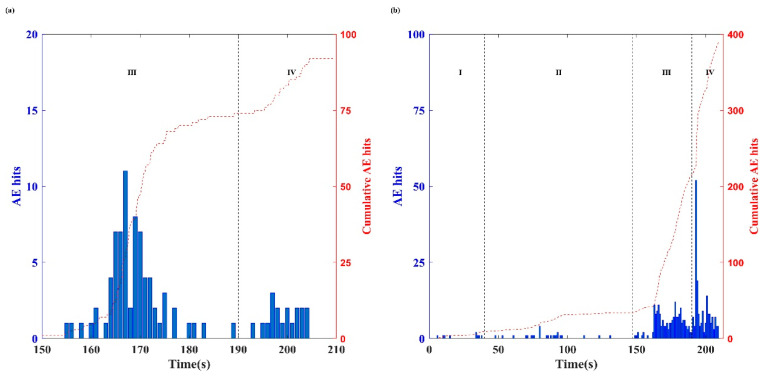
The variations in AE hits and cumulative AE hits of two types of the AE signals: (**a**) burst signals, and (**b**) continuous signals.

During the failure process of the specimens, multiple AE signals are released every second. To explore the internal relationship between AE hits and the damage of the locking section, the evolution characteristics of AE hits and cumulative AE hits of the two types of signals are analyzed. AE hits represent the number of AE signals released per second, related to the number of cracks [24]. Cumulative AE hits denote the cumulative sum of signals up to the current moment, reflecting its growth trend [45]. Figure 8 illustrates the variations in AE hits over time in the test, depicting both instantaneous AE hits and cumulative AE hits, which can be described in detail as follows.

(1) In the stage of compaction by initial damage (stage I), the cumulative AE hits of the continuous signal gradually increased, reflecting the closure of pores and cracks in the rock caused by the stress.
(2) In the elastic deformation stage (stage II), AE hits from the continuous signal remained at a low level, and the increase rate of the cumulative AE hits gradually stabilized. AE inactivity implies the rock grains reached a state of balance and associated with accumulating energy inside the locking section.
(3) In the crack propagation and failure stages (stages III and IV), a burst signal first appeared at 147.8 s, indicating the formation of microcracks within the locking section. Therefore, in this study, the initial appearance of the burst signal was also used as the first early-warning point obtained from the AE hits. The emergence of the burst signal provides critical information obtained exclusively from the classification results. Accordingly, the time of the first early-warning point obtained from the AE hits (147.8 s) is 15.2 s earlier than that obtained from the MS signal (163 s). From 147.8 to 163 s, the AE hits increased slowly, indicating steady initiation and propagation of microcracks. Then, the cumulative AE hits of burst and continuous signals increased sharply induced by the shear failure of the locking section and microcracks were coalesced and propagated unstably. Therefore, the rapid increase in cumulative AE hits was regarded as the second early-warning point obtained from the AE hits, and the time of the second early-warning key point obtained from the AE hits is consistent with that of the MS signal, which is 163 s. After the shear failure occurred in the front and back of the locking segment alternately, the number of AE hits and cumulative AE hits increased again at 193 s, indicating that the main crack was forming as well as the sudden brittle shear failure of the locking section. It is worth noting that the number of AE hits of burst signal shows a decreasing trend, while the number of AE hits of continuous signal continues to remain at a high level.

### 4.2. Maximum Amplitude of Waveform (Intensity)

**Figure 9:**
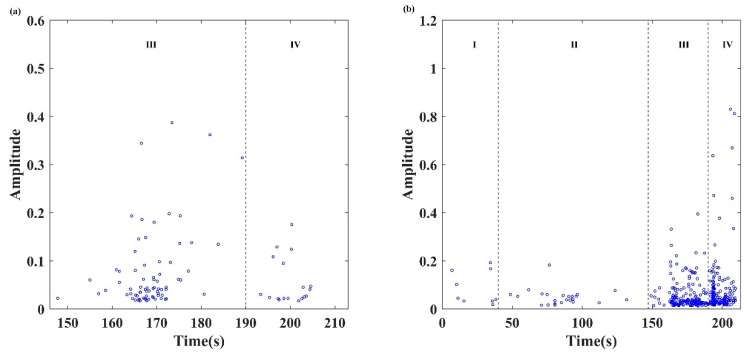
The distribution of the maximum amplitude of AE waveform: (**a**) burst signals, and (**b**) continuous signals.

The amplitude of the AE waveform can reflect the intensity of the AE event. The process of microcrack initiation, propagation, and gradual convergence in the rock led to low amplitude. The increasing amplitude means that more and more microcracks develop and fuse to form large-scale cracks [46]. In this study, by calculating the maximum value of each waveform data, the maximum amplitude of the two types of AE signals during the failure process was obtained, as shown in Figure 9. The evolutionary process of the maximum amplitude is described as follows.

(1) In the stage of compaction by initial damage (stage I), some continuous signals with low amplitude can be observed due to the compaction of internal pore.
(2) In the elastic deformation stage (stage II), the load level is insufficient to induce new microcracks inside the locking section. AE events were caused by the occlusal failure of rough surfaces when cracks caused a relative slip. Therefore, a small number of continuous signals with low energy were observed.
(3) In the stage of cracks propagation and failure (stages III and IV), the amplitude of the burst signals gradually increased and then decreased, while the amplitude of the continuous signals increased monotonously. The above phenomenon implies that several microcracks appeared with low energy after the main crack appeared, and continuous signals with higher energy were caused by the occlusal failure of rough surfaces when the cracks caused relative slippage.

### 4.3. Frequency

AE signals are characterized as non-stationary signals, and FFT is a classical spectrum analysis method to analyze the non-stationary signal. FFT was carried out for each of the AE data to extract the main frequency and sub-frequency of the corresponding waveform [40]. In this section, we analyze the frequency characteristics of burst signal and continuous signal, respectively.

#### 4.3.1. Frequency Characteristics of Burst Signals

**Figure 10:**
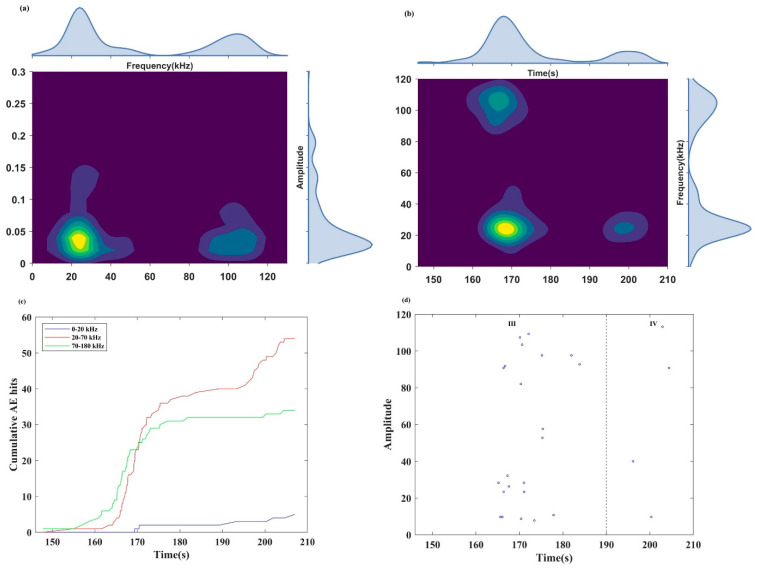
The characteristics of burst signals: (**a**) the main frequency–amplitude distribution; (**b**) the distribution of the main frequency; (**c**) the distribution of the cumulative AE hits in each main frequency band; and (**d**) the sub-frequency distribution.

Figure 10 shows the frequency distribution of burst signals, including the main frequency and sub-frequency. The frequencies were in the range from 0 to 180 kHz. Based on the frequency distribution, the entire frequency band was divided into three bands: 0–20 kHz (low-frequency band), 20–70 kHz (mid-frequency band), and 70–180 kHz (high-frequency band). The main frequency–amplitude characteristics of burst signals are presented in Figure 10a. It is observed that the main frequencies of burst signals were all distributed in the high- and middle-frequency bands, and there were relatively few low-frequency burst signals. Furthermore, high-amplitude burst signals were predominantly concentrated in the high- and middle-frequency bands.

The evolution characteristics of the main frequency and the cumulative AE hits in each of the main frequency bands of burst signals are shown in Figure 10b and c, respectively.

Before 147.8 s, the signal has no significant change in characteristics. From 147.8 to 163 s, the cumulative AE hits in the high-frequency band of the burst signals increased from 1 to 7, while the cumulative AE hits in the other frequency bands remained relatively stable. According to Cai et al. [47], high-frequency AE signals correspond to the initiation of microcracks, and the low-frequency signals correspond to the formation of large cracks. Therefore, the appearance of high-frequency AE signals indicates the occurrence of microcracks inside the locking segment and is the early-warning point of rock instability. The high-frequency burst signal was detected at 147.8 s, so the time of the early-warning point obtained from the frequency of burst signals (147.8 s) is 15.2 s earlier than that obtained from the MS signal (163 s). The microcracks were coalesced and propagated stably until 163 s.

From 163 to 180 s, burst signals began to appear in large numbers and were concentrated in the middle and high frequency; the cumulative AE hits in the middle- and high-frequency bands increased rapidly. The main frequency showed a penetrating trend in the whole frequency band, indicating that microcracks were rapidly generated, and the microcracks began to propagate to a few large cracks, to coalescence, and to form macrocracks.

From 180 to 190 s, the increase rate of the cumulative AE hits of burst signals in the middle- and high-frequency bands decreased gradually. After the main crack occurred, the increased rate of the cumulative AE hits in high frequency decreased obviously, indicating there were fewer microcracks in the specimen.

Figure 10d shows the sub-frequency characteristics of the burst signals. Burst signals in each frequency band have scattering characteristics in crack propagation and fracture stage (stages III and IV).

#### 4.3.2. Frequency Characteristics of Continuous Signals

**Figure 11:**
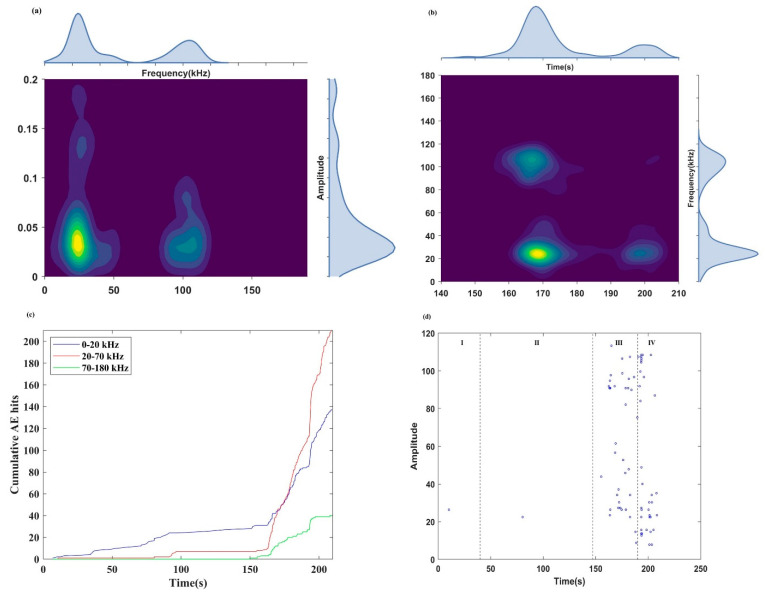
The characteristics of continuous signals: (**a**) the main frequency–amplitude distribution; (**b**) the distribution of the main frequency; (**c**) the distribution of the cumulative AE hits in each main frequency band; and (**d**) the sub–frequency distribution.

Figure 11 shows the frequency distribution of continuous signals. Like the burst signal, the frequency band is divided into three parts. Figure 11a presents the main frequency–amplitude distribution of continuous signals. The main frequency–amplitude distribution of continuous signals is significantly different from that of burst signals. Continuous signals were mainly distributed in the low- and middle-frequency bands, with high-amplitude continuous signals predominantly found in the low-frequency band.

Figure 11b and c, respectively, show the evolution process of the main frequency and the cumulative AE hits in each main frequency band of the continuous signals.

Only a few continuous signals with low frequency were received during the initial compaction stage and elastic stage (stages I and II). In the stage of cracks propagation (stage III), a high-frequency continuous signal appeared for the first time at 150.9 s, marking the early-warning point of rock instability. And the time of the early-warning point obtained from the frequency of continuous signals (150.9 s) is 12.1 s earlier than that obtained from the MS signal (163 s) but 3.1 s later than that obtained from the frequency of burst signals (147.8 s). The microcracks coalesced and propagated stably from 150.9 to 163 s.

From 163 to 193 s, the cumulative AE hits of continuous signals in the low- and middle-frequency bands increased at a high rate. In contrast, the cumulative AE hits of high-frequency continuous signals increased slowly, indicating that the microcracks spread and penetrated to form large-scale ruptures at this time. The growth rate of the cumulative AE hits in 193 s was the highest, and the cumulative AE hits in each frequency band shows a sudden increase trend again, indicating that the main crack was about to form.

The main crack caused relative slippage at 198 s, and the cumulative AE hits of the continuous signals in low- and medium-frequency bands increased steadily, but the cumulative AE hits of the continuous signals in the high-frequency band remained almost stable, indicating that a small amount of small-scale fracture occurred at this time.

According to the evolution law of the sub-frequency in Figure 11d, the sub-frequency mainly appeared in the stage of cracks propagation and failure (stages III and IV), and the sub-frequencies in the low-frequency band were only detected in the stage of rock instability and failure (stage IV), indicating that the sub-frequency migrated to a low frequency.

## 5. Discussion

### 5.1. Evolution Characteristics of Sample #1

In the stage of compaction by initial damage (stage I), the internal cracks and voids in the locking section of the three-section model were compacted and closed. The amplitude of the MS signal decreased gradually. Frequency components of the MS signal were in the range from 250 to 500 Hz, and then migrated to a low frequency. Meanwhile, a small number of AE hits occurred, primarily continuous signals with low amplitude and low frequency.

In the elastic deformation stage (stage II), the amplitude of the MS signal remained at a low level. The main frequencies of the MS signals were almost all distributed below 250 Hz. Relatively few continuous signals with low amplitude and low frequency were detected at the same time.

In the stage of cracks propagation and failure (stages III and IV), the high-frequency burst signal appeared for the first time at 147.8 s, indicating that the locking section was sheared and microcracks inside the locking section occurred, and the high-frequency continuous signal was detected for the first time at 150.9 s. The cumulative AE hits of the AE signals increased rapidly at 163 s, indicating the rapid expansion of the microcracks in the locking section. Concurrently, the high-frequency components of the MS signal appeared again. All of these are indicators as early-warning points. From 163 to 190 s, numerous burst signals with higher amplitudes were noted in the middle- and high-frequency bands. Meanwhile, a substantial number of low-amplitude continuous signals appeared in low- and middle-frequency bands. From 190 to 212 s (stage IV), low-amplitude burst signals in the mid-frequency band occurred, and several high-amplitude continuous signals in the low-frequency band were detected.

**Figure 12:**
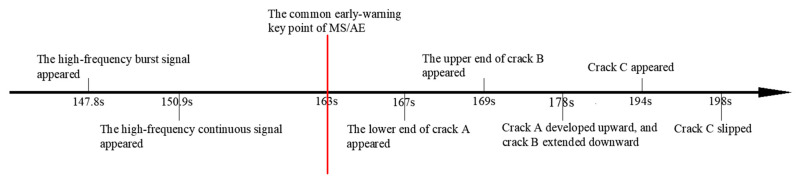
The time axis of the early-warning points and macrocracks propagation of sample #1.

In summary, the above analysis reveals the time axis of the early-warning key points and crack propagation. As shown in Figure 12, the MS signal, burst signal, and continuous signal all exhibited one early-warning point at 163 s, which was 4 s earlier than the appearance of a macroscopic crack (derived from video image). Comparative analysis indicates that the early-warning points of AE signals preceded those of the MS signal, with the MS signal showing only one early-warning point. The appearance of a burst signal is the key warning information of sample rupture and instability, and its time was earlier than the time of the early-warning point of the overall AE signals. The first appearance of high-frequency AE signals indicates the occurrence of microcracks, so it can be used as beneficial information for prediction. Notably, the high-frequency burst signal first appeared at 147.8 s, 3.1 s earlier than the continuous signal.

### 5.2. Evolution Characteristics of Other Samples

**Figure 13:**
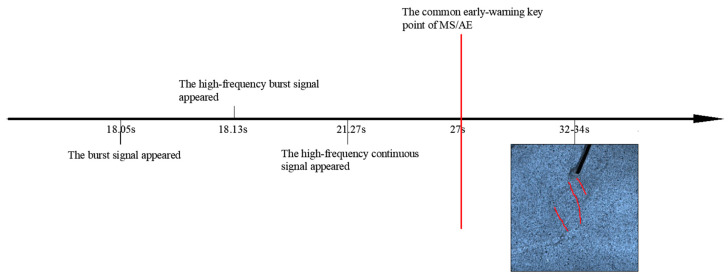
The time axis of the early-warning points and macrocracks propagation of sample #2. The red lines in picture indicates the macrocracks.

**Figure 14:**
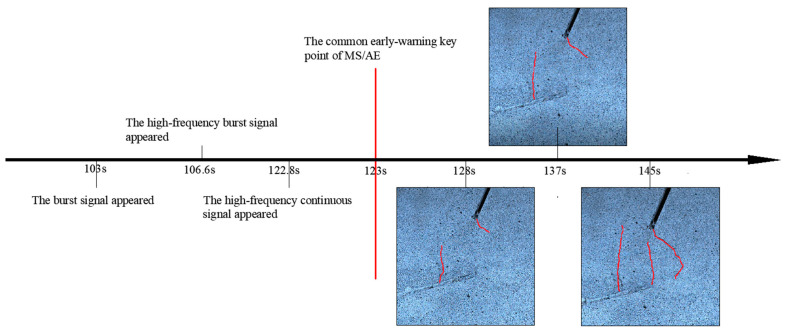
The time axis of the early-warning points and macrocracks propagation of sample #3. The red lines in picture indicates the macrocracks.

Based on the above processing procedure, the test data of samples #2 and #3 were processed. The time axis of the early-warning points and crack propagation are shown in Figure 13 and Figure 14. It is evident from the figures that the MS signal and AE signals respond to the early-warning point earlier than the video image. Importantly, the early-warning points of AE signals preceded those of the MS signals. It is worth noting that the time of the early-warning point of the AE signals was earlier than the MS signals. Among AE signals, the time of the early-warning point of the burst signal was earlier than the continuous signal. The time of the early-warning point of the classification results was earlier than the time of the overall AE signals. These findings align consistently with those observed in sample #1.

## 6. Conclusions

This study utilizes video imaging, MS signal, and AE signal to analyze the evolution characteristics and early-warning indicators of three-section landslides. We proposed a novel method to classify AE signals into burst signals and continuous signals, studying the features of the two types, respectively. The findings of this study are summarized as follows:

(1) Pronounced distinctions are evident between burst signals and continuous signals concerning event frequency, intensity, and activity. Continuous signals exhibited significantly higher total AE hits compared to burst signals, which were exclusively observed during the crack propagation and failure stage. High-amplitude burst signals were predominantly distributed in the middle- and high-frequency bands, while high-amplitude continuous signals were primarily distributed in the low-frequency band. During the crack propagation stage, both burst signals and continuous signals with high amplitude occurred. In the stage of rock instability and failure, the AE hits of burst signals exhibited a decreasing trend, with their amplitude gradually declining, while the AE hits of the continuous signal remained at a high level, with further amplitude increases.
(2) The emergence of a burst signal indicates the occurrence of microcracks in the rock mass, serving as an early-warning point of rock instability. Notably, this critical information is only obtained from the classification results. The generation of high-frequency AE signals indicates the presence of microcracks in the specimen, which can propagate and converge into macrofractures. Therefore, the occurrence of high-frequency AE signals is employed as an early-warning point for rock instability.
(3) Comparison of the timing of the early-warning points derived from the video imaging, MS signals, and AE signals revealed that both MS signals and AE signals preceded video imaging, with AE signals leading MS signals. The occurrence of early-warning points was more frequent with AE signals compared to MS signals. Importantly, the timing of the early-warning point for burst signals preceded that of continuous signals, and the timing of the early-warning point for the classification results preceded that of the overall AE signals. The research findings indicate that AE and MS are more suitable as precursor warnings for sudden landslide disasters than deformation monitoring indicators.

## Figures and Tables

**Table 1:** Proportions of the number of AE signals in each stage.

Proportions (%)	Stage I	Stage II	Stage III	Stage IV	Whole Process
Burst signals	0.00	0.00	15.32	3.93	19.25
Continuous signals	1.86	5.18	37.27	36.44	80.75
AE signals	1.86	5.18	52.59	40.37	100.00

## Data Availability

The data that support the findings of this study are available from the corresponding author upon reasonable request.
